# Current Approach of Functioning Head and Neck Paragangliomas: Case Report of a Young Patient with Multiple Asynchronous Tumors

**DOI:** 10.1155/2020/6827109

**Published:** 2020-01-30

**Authors:** Alejandro Terrones-Lozano, Alan Hernández-Hernández, Edgar Nathal Vera, Gerardo Yoshiaki Guinto-Nishimura, Jorge Luis Balderrama-Bañares, Claudia Ramírez-Rentería, Judith de la Serna-Soto, Alfredo Adolfo Reza-Albarran, Lesly Portocarrero-Ortiz

**Affiliations:** ^1^Instituto Nacional de Neurología y Neurocirugía Manuel Velasco Suárez (INNN) Neuroendocrinology, Mexico City, Mexico; ^2^Neurosurgery (INNN), Mexico City, Mexico; ^3^Endovascular Therapy (INNN), Mexico City, Mexico; ^4^Unidad de Investigación en Endocrinología Experimental, Siglo XXI Instituto Mexicano del Seguro Social, Mexico City, Mexico; ^5^Neuroanesthesia (INNN), Mexico City, Mexico; ^6^Instituto Nacional de Ciencias Medicas y Nutrición, Mexico City, Mexico

## Abstract

*Introduction*. Pheochromocytomas (Pheo) and paragangliomas (PGL) are rare neuroendocrine tumors arising from chromaffin cells of the adrenal medulla and from the extra-adrenal autonomic paraganglia, respectively. Only 1–3% of head and neck PGL (HNPGL) show elevated catecholamines, and at least 30% of Pheo and PGL (PCPG) are associated with genetic syndromes caused by germline mutations in tumor suppressor genes and proto-oncogenes. *Clinical Case*. A 33-year-old man with a past medical history of resection of an abdominal PGL at the age of eleven underwent a CT scan after a mild traumatic brain injury revealing an incidental brain tumor. The diagnosis of a functioning PGL was made, and further testing was undertaken with a PET-CT with 68Ga-DOTATATE, SPECT-CT 131-MIBG, and genetic testing. *Discussion and Conclusion*. The usual clinical presentation of functioning PCPG includes paroxistic hypertension, headache, and diaphoresis, sometimes with a suggestive family history in 30–40% of cases. Only 20% of PGL are located in head and neck, of which only 1–3% will show elevated catecholamines. Metastatic disease is present in up to 50% of cases, usually associated with a hereditary germline mutation. However, different phenotypes can be observed depending on such germline mutations. Genetic testing is important in patients with PCPG since 31% will present a germline mutation. In this particular patient, an *SDHB* gene mutation was revealed, which can drastically influence the follow-up plan and the genetic counsel offered. A multidisciplinary approach is mandatory for every patient presenting with PCPG.*SDHB* gene mutation was revealed, which can drastically influence the follow-up plan and the genetic counsel offered. A multidisciplinary approach is mandatory for every patient presenting with PCPG.

## 1. Introduction

Pheochromocytomas (Pheo) and paragangliomas (PGL) are rare neuroendocrine tumors arising from chromaffin cells of the adrenal medulla and from extra-adrenal autonomic paraganglia, respectively, with an estimated incidence of 0.8 cases per 100,000 person-years [1]. Pheo and sympathetic PGL are almost always functional (i.e., clinically active) while parasympathetic PGL are usually not [2], since only 1–3% of head and neck PGL (HNPG) show elevated catecholamines. At least 30% of Pheo and PGL (PCPG) are associated with germline mutations in certain susceptibility genes [2, 3]. However, different mutations may display different phenotypes, including tumor type, location, frequency, risk of malignancy, and heritability pattern. Since genetic testing is expensive and not widely available in most countries, prioritizing genetic testing requires a careful clinical and biochemical evaluation, which can significantly reduce costs and improve health care [4]. Functional imaging modalities may also be useful adjuncts for both diagnosis and workup for metastatic disease. Somatostatin receptor scintigraphy has been recently proposed as a useful method for localizing PGL, particularly in the head and neck [5]. We hereby present a case of head and neck catecholamine producing PGL, emphasizing on the importance of an individualized approach by a multidisciplinary team to guide diagnosis, treatment, and surveillance protocols based on current management recommendations.

## 2. Case Report

A 33-year-old man underwent a CT scan after a mild traumatic brain injury. The study revealed an incidental brain mass, which was initially considered to be a meningioma. The patient was referred to the National Institute of Neurology and Neurosurgery for surgical treatment. His family history was unremarkable for oncologic diseases. His past medical history as recalled by the patient included the resection of an abdominal para-aortic PGL at 11 years of age and a Pheo at 19 years of age. At that time, the patient was living abroad, where he was evaluated and treated, but his medical records were not available after request at the time of his referral to our center. During his childhood, he was initially evaluated because he had clinical symptoms of intermittent claudication, chest pain associated with exertion, severe hypertension, tachycardia, and excessive sweating. After a PGL was diagnosed, he was subject to a complete tumor resection and remained asymptomatic for 8 years, when the routine follow-up examination showed a marked elevation of catecholamines. A new imaging study was made, which revealed a right adrenal gland pheochromocytoma, which was also surgically removed, becoming once again asymptomatic for several years, after which he lost follow-up in that facility. During that time, he moved to Mexico, where he continued having an apparently normal life without any further medical evaluation for the next 14 years, up until the event of the head trauma.

**Figure 1:**
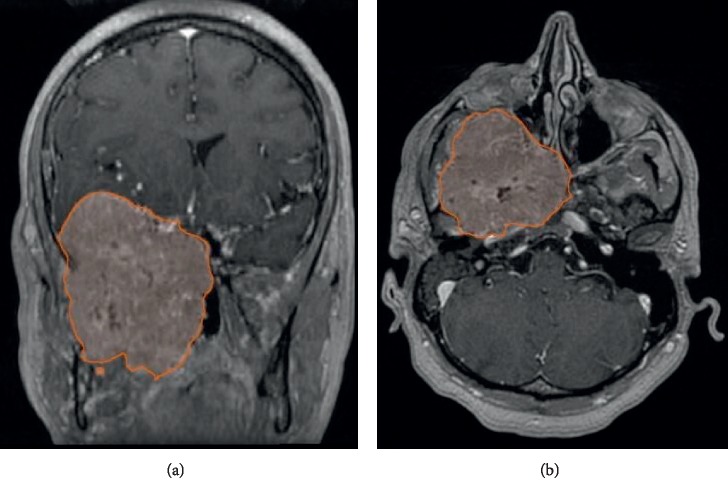
T1 Enhanced-MRI: tumor lesion located at the right skull base with 163.2 cm^3^ volume.

**Figure 2:**
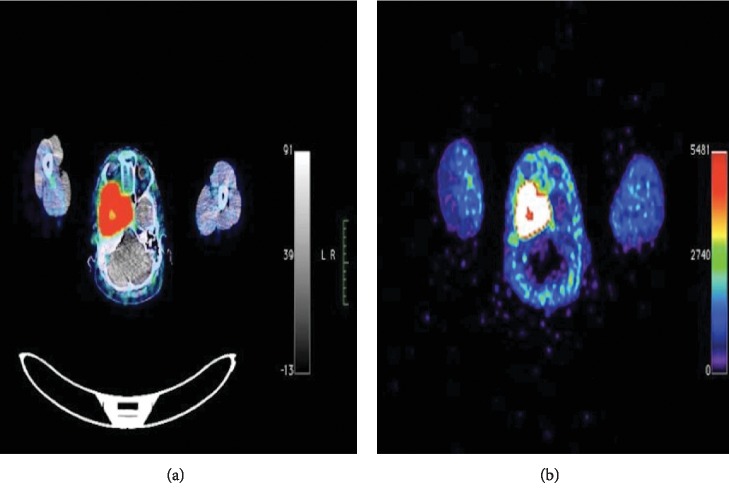
Transversal image from the full body PET-CT scan with 68GA-DOTATATE (a) and 131-MIBG SPECT (b) showing intense uptake in the skull base tumor.

During our initial evaluation, the patient was found to have right moderate hearing loss, right proptosis, optic nerve atrophy with right temporal hemianopsia, and face hyperesthesia in the area corresponding to the V2 and V3 branches of the trigeminal nerve. Blood pressure was reported to be intermittently high, between 130/90 and 140/100 mmHg. No weight changes, fatigue, nausea, hair loss, or other hormone deficiency-related symptoms were referred by the patient after purposely inquiring. The MRI was initially reported to be compatible with a meningioma vs. a hemangiopericytoma; however, the medical history called for additional diagnostic workup (Figure 1). Laboratory results showed normal metanephrines 208 *μ*g (normal range 86–320 *μ*g/24 H) and elevated normetanephrines 10,828 *μ*g (normal range 120–400 *μ*g/24 H; 27 times the upper limit of normal); the rest of the hormonal tests were normal. The diagnosis of a functioning PGL was then established, but the low incidence of functioning HNPG as well as the apparently aggressive behavior lead us to request a positron-emission tomography-computed tomography (PET-CT) with 68Ga-DOTATATE, which showed an increased expression of somatostatin receptors in the lesion (Figure 2). The single-photon emission computed tomography with metaiodobenzylguanidine (SPECT-CT 131-MIBG) was also positive in the skull base lesion, related to an abnormal synthesis of catecholamines (Figure 2).

The patient was started on medical preoperative management with selective alpha-blockade (Prazosin) in a 3 mg three times a day dose and nifedipine 30 mg daily for two weeks, with the systolic blood pressure being lowered to 130/80 mmHg, when he was started on beta-blockers (Atenolol) 50 mg twice a day for an additional six weeks. In order to reduce excessive bleeding risk during open surgery, the decision was made to perform an endovascular preoperative embolization of the right maxillary artery using polyvinyl alcohol microspheres. Despite adequate preoperative medical treatment, the patient suffered from alternating episodes of bradycardia and supraventricular tachycardia, as well as hypertensive crisis during the procedure, when only 40% of the tumor been successfully embolized. The endovascular procedure was stopped, and an emergency MRI was performed. The image showed signs of increased intracranial pressure without any areas of hemorrhage or ischemia; however, since the patient had an altered mental status and bilateral pupillary dilation, a decompressive hemicraniectomy was emergently performed. After a successful intervention, he was transferred to the intensive care unit (ICU) for recovery.

**Figure 3:**
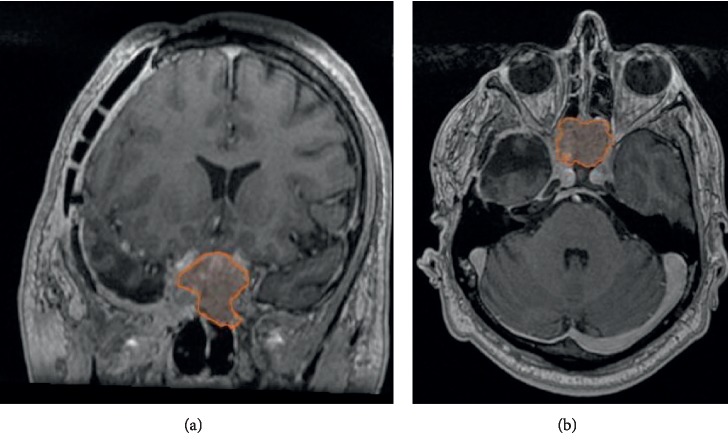
Follow-up MRI with residual tumor lesion near the sellar region extending to nasal cavity with 11.9 cm^3^ volume.

After 20 days from the initial procedure, the patient was scheduled for a second embolization procedure and tumor resection surgery. After the second embolization, 95% of the tumor was successfully embolized without any complications, and 48 hours, later the patient underwent surgery achieving a subtotal tumor resection (Figure 3). He had an excellent postoperative recovery period with normalization of previously elevated catecholamines and improvement of his visual field, and was discharged home without further complications. He has now been followed-up for an additional 2 years. His blood pressure is normal without any medication, his urinary and serum catecholamines are normal, and the tumor remnant has not increased in follow-up MRI scans (Figure 3).

The genetic testing showed a heterozygous *SDHB* gene mutation, reference sequence; NM_003000.2: c.136C>T, NP_002991.2: p.Arg46^*∗*^ (clinvar: RCV000505277.1), which has been reported to be a pathogenic variant.

## 3. Discussion

PCPG are neuroendocrine tumors arising from chromaffin cells, which can produce catecholamines. The most common location is the adrenal glands (Pheo), while extra-adrenal tumors are called PGL [5]. The classical clinical presentation of functioning PCPG includes paroxistic hypertension, headache, and diaphoresis; a positive family history may be found in 30–40% of cases. These lesions can be found incidentally in 5% of cases [6]. Even though most HNPGs are clinically silent, some of them are actively synthesizing catecholamines but degrading them quickly inside the tumor tissue, without any biochemically or clinically significant secretion into the bloodstream under normal conditions. However, since these peptides may be released during surgical procedures or manipulation, planning an optimal preoperative medical management strategy with a multidisciplinary team is mandatory [7, 8]. The treatment of choice is a complete surgical resection, which may be difficult to achieve depending mainly on tumor size and location, as well as the team's surgical experience. For this reason, endovascular embolization has been extensively described in the management of several head and neck vascularized tumors including PGL [9]. Even though preoperative embolization has proven to reduce blood loss and operative time in the resection of most HNPG [10, 11], some particular indications have not been clearly defined, as in the case of carotid body tumors [12, 13]. However, in other types of PGL such as in this case, preoperative embolization has proven to be a useful adjunct to improve surgical conditions when performed appropriately by an experienced team [9, 14, 15]. Cranial base PGL may be an even stronger indication due to the relatively difficult access to this location [12]. It is important to consider the risk of cranial nerve palsies and other complications after preoperative embolization so that a correct decision can be made regarding the appropriate treatment for these patients.

Patients presenting at a young age, with multiple endocrine tumors, either synchronous or asynchronous, a positive family history, or other syndromic manifestations have an increased risk of having an underlying genetic mutation. Regardless, current recommendations suggest that all patients diagnosed with PCPG should be considered for genetic testing, since up to 30% of apparently sporadic cases may be associated to a germline mutation [16]. Different phenotypes, including location, size, risk of malignancy, and other associated tumors, have been linked to individual germline mutations. Based on these differences, particular management and surveillance guidelines have been proposed according to the genetic profile. [17] Approximately 20% of PGL are located in the head and neck, representing around 0.6% of all head and neck tumors and 0.03% of all tumors [7]. However, certain germline mutations may be associated with a significant predisposition for HNPG, with an estimated risk of 30% in cases of *SDHB* mutations but up to 79% in *SDHD* mutations [16–18]. Germline mutations in the *SDHB* gene are transmitted in an autosomal dominant pattern with an incomplete penetrance. The mean age at diagnosis is approximately 30 years, similar as in other germline mutations [19]. The penetrance of SDHB mutations varies widely but is generally lower than that of *SDHD* or *SDHAF2* mutations, with reported values of 20% at 50 years and 40% at 70 years of age [20, 21]. Tumors associated to this mutation occur most commonly at thoracic or abdominal extra-adrenal locations, but HNPG may be present in 20–30% of cases. Even though multifocal disease is not as frequent as in the case of other mutations (such as *SDHD*), *SDHB* mutations carry an increased risk for malignant transformation of PCPG as well as other malignant tumors such as renal cell carcinoma or thyroid carcinoma [16, 17, 19, 22]. In addition to the *SDHB* mutation status, several risk factors have been identified for malignant transformation of PGL as well as for overall survival in these patients, such as age at diagnosis, tumor size, and presence of synchronous metastases [1, 23–25]. In the present study, we describe the case of a patient with an initial diagnosis of a PGL during the pediatric age that developed other asynchronous and functioning tumors during the following 22 years. His initial age at diagnosis increases the chance of having a genetic mutation to >40%, while the biochemical profile and tumor location suggested that an *SDHB* gene mutation might be the culprit [22]. The clinical manifestations of parasympathetic-derived tumors arise mainly due to their large size and compressive effects such as hearing loss and cranial nerve palsy. Studies suggest that up to 41% of patients with functioning PGL have a germline mutation in one of the known common susceptibility genes (including *NF1*, *VHL*, *RET*, *SDHB*, *SDHD*, and *SDHC*). Up to fifty-three percent of patients coursing with at least one extra-adrenal PGL have an identified mutation [22, 26]. In this particular case, differential diagnosis must be established with an *SDHD* gene mutation, which is also associated with PCPG but present a rather different phenotype [27]. As in this case, the most commonly mutated gene is *SDHB*, which carries the highest risk of malignancy (up to 40%) and therefore present a poor prognosis [27]. The identification of a germline mutation in the *SDHB* gene demands close surveillance since these patients have a 4.7% risk of developing renal cell carcinoma by the age of 60 [26, 27]. A genetic test result will help us establish an adequate follow-up plan for the patient and his relatives to offer a more precise genetic counsel considering his prognosis and future quality of life.

At his age, he has already undergone four surgeries, and the cardiovascular and anesthetic risks have been increasing, not to mention the risk of malignancy or regrowth since the primary cranial tumor was not completely resected. Genetic counseling is also important in these patients in the long term due to the association of other endocrine and nonendocrine tumors [22, 27]. Close follow-up must be established in this patient in order to detect any tumor recurrence or other de novo neoplasms associated with his genetic condition. Current recommendations suggest that carriers of these mutations need to be screened for tumors beginning at age 5 at 6- to 12- months intervals. Biochemical testing (urinary or plasma metanephrines) is suggested annually, an anatomical imaging study (MRI or CT scan of the abdomen, thorax, and pelvis and skull base/neck) every 2 years, with further consideration of a functional imaging test (MIBG scintigraphy, PET-CT scan) [28]. Screening for renal cell carcinoma should also be considered [29]. However, follow-up must be individually tailored in every case, especially when malignant or remnant tumors are suspected or when clinical symptoms are present [30]. A successful transition from pediatric to adult care is mainstay for an adequate follow-up of these patients. In this case, the patient was lost to follow-up for several years without any medical evaluation, despite the fact that he had been closely monitored during his pediatric years. Apparently, neither the patient nor his family remember being told about the possibility of a new tumor later in life and they did not keep any documents from his consult visits, which would have increased our diagnostic suspicion and expedited the management. Given the cost of genetic testing, the family members have declined evaluation, which could also be a great limitation for an adequate treatment in many emerging economies.

## 4. Conclusion

The present case adequately highlights several important aspects to consider during the diagnostic workup and management of patients with PCPG. A complete family history and a detailed past medical history are necessary to raise suspicion about a certain disease pattern, while the appropriate laboratory and imaging studies are useful adjuncts to confirm the diagnosis. Preoperative management must be tailored to each patient and carefully considered since it may influence outcomes as much as the procedure itself. Even though there are risk factors associated to a germline mutation, genetic testing should be considered in all patients with PCPG in order to plan accordingly the appropriate follow-up plan and to offer adequate genetic counsel to the patients and their relatives. A multidisciplinary team must be actively involved in every aspect of the patient's care in order to enhance an adequate decision-making process.

## References

[1] Hamidi O., Young W. F., Iñiguez-Ariza N. M. (2017). Pheochromocytoma and paraganglioma: 272 patients over 55 years. *The Journal of Clinical Endocrinology and Metabolism*.

[2] Welander J., Söderkvist P., Gimm O. (2011). Genetics and clinical characteristics of hereditary pheochromocytomas and paragangliomas. *Endocrine-Related Cancer*.

[3] Offergeld C., Brase C., Yaremchuk S. (2012). Head and neck paragangliomas: clinical and molecular genetic classification. *Clinics*.

[4] Neumann H. P. H., Erlic Z., Boedeker C. C. (2009). Clinical predictors for germline mutations in head and neck paraganglioma patients: cost reduction strategy in genetic diagnostic process as fall-out. *Cancer Research*.

[5] Hubalewska-Dydejczyk A., Timmers H. J. L. M., Trofimiuk-Müldner M. (2015). The place of somatostatin receptor scintigraphy and other functional imaging modalities in the setting of pheochromocytoma and paraganglioma. *Somatostatin Analogues*.

[6] Kasperlik-Zaluska A. A., Roslonowska E., Slowinska-Srzednicka J. (2006). 1,111 patients with adrenal incidentalomas observed at a single endocrinological center: incidence of chromaffin tumors. *Annals of the New York Academy of Sciences*.

[7] Fishbein L. (2016). Pheochromocytoma and paraganglioma. *Hematology/Oncology Clinics of North America*.

[8] Li J., Yang C. H. (2014). Improvement of preoperative management in patients with adrenal pheochromocytoma. *International Journal of Clinical and Experimental Medicine*.

[9] Duffis E. J., Gandhi C. D., Prestigiacomo C. J. (2012). Head, neck, and brain tumor embolization guidelines. *Journal of NeuroInterventional Surgery*.

[10] White J. B., Link M. J., Cloft H. J. (2008). Endovascular embolization of paragangliomas: a safe adjuvant to treatment. *Journal of Vascular and Interventional Neurology*.

[11] Jackson R. S., Myhill J. A., Padhya T. A., McCaffrey J. C., McCaffrey T. V., Mhaskar R. S. (2015). The effects of preoperative embolization on carotid body paraganglioma surgery. *Otolaryngology-Head and Neck Surgery*.

[12] Michelozzi C., Januel A. C., Cuvinciuc V. (2016). Arterial embolization with Onyx of head and neck paragangliomas. *Journal of NeuroInterventional Surgery*.

[13] Abu-Ghanem S., Yehuda M., Carmel N. N., Abergel A., Fliss D. M. (2016). Impact of preoperative embolization on the outcomes of carotid body tumor surgery: a meta-analysis and review of the literature. *Head & Neck*.

[14] Persky M. S., Setton A., Niimi Y., Hartman J., Frank D., Berenstein A. (2002). Combined endovascular and surgical treatment of head and neck paragangliomas? A team approach. *Head & Neck*.

[15] Moore M. G., Netterville J. L., Mendenhall W. M., Isaacson B., Nussenbaum B. (2016). Head and neck paragangliomas. *Otolaryngology-Head and Neck Surgery*.

[16] Muth A., Crona J., Gimm O. (2019). Genetic testing and surveillance guidelines in hereditary pheochromocytoma and paraganglioma. *Journal of Internal Medicine*.

[17] Neumann H. P. H., Pawlu C., Peczkowska M. (2004). Distinct clinical features of paraganglioma syndromes associated with SDHB and SDHD gene mutations. *JAMA*.

[18] Guha A., Musil Z., Vicha A. (2019). A systematic review on the genetic analysis of paragangliomas: primarily focused on head and neck paragangliomas. *Neoplasma*.

[19] Neumann H. P., Young W. F., Krauss T. (2018). 65 years of the double helix: genetics informs precision practice in the diagnosis and management of pheochromocytoma. *Endocrine-Related Cancer*.

[20] Rijken J. A., Niemeijer N. D., Jonker M. A. (2018). The penetrance of paraganglioma and pheochromocytoma in SDHB germline mutation carriers. *Clinical Genetics*.

[21] Hensen E. F., Jordanova E. S., van Minderhout I. J. (2004). Somatic loss of maternal chromosome 11 causes parent-of-origin-dependent inheritance in SDHB-linked paraganglioma and pheochromocytoma families. *Oncogene*.

[22] Andrews K. A., Ascher D. B., Pires D. E. V. (2018). Tumor risks and genotype-phenotype correlations associated with germline variants in succinate dehydrogenase subunit genes SDHB, SDHC and SDHD. *Journal of Medical Genetics*.

[23] Sutton M. G., Sheps S. G., Lie J. T. (1981). Prevalence of clinically unsuspected pheochromocytoma. Review of a 50-year autopsy series. *Mayo Clinic Proceedings*.

[24] Eisenhofer G., Lenders J. W., Siegert G. (2012). Plasma methoxytyramine: a novel biomarker of metastatic pheochromocytoma and paraganglioma in relation to established risk factors of tumour size, location and SDHB mutation status. *European Journal of Cancer*.

[25] Schovanek J., Martucci V., Wesley R. (2014). The size of the primary tumor and age at initial diagnosis are independent predictors of the metastatic behavior and survival of patients with SDHB-related pheochromocytoma and paraganglioma: a retrospective cohort study. *BMC Cancer*.

[26] Fishbein L., Merrill S., Fraker D. L., Cohen D. L., Nathanson K. L. (2013). Inherited mutations in pheochromocytoma and paraganglioma: why all patients should be offered genetic testing. *Annals of Surgical Oncology*.

[27] Jochmanova I., Wolf K. I., King K. S. (2017). SDHB-related pheochromocytoma and paraganglioma penetrance and genotype-phenotype correlations. *Journal of Cancer Research and Clinical Oncology*.

[28] Eijkelenkamp K., Osinga T. E., de Jong M. M. (2017). Calculating the optimal surveillance for head and neck paraganglioma in SDHB-mutation carriers. *Familial Cancer*.

[29] Ricketts C. J., Forman J. R., Rattenberry E. (2010). Tumor risks and genotype-phenotype-proteotype analysis in 358 patients with germline mutations in SDHB and SDHD. *Human Mutation*.

[30] Lerebvre M., Foulkes W. D. (2014). Pheochromocytoma and paraganglioma syndromes: genetics and management update. *Current Oncology*.

